# Restlessness in cluster headache: unveiling the role of its key associations and clinical implications from a cohort study

**DOI:** 10.3389/fneur.2026.1946721

**Published:** 2026-10-05

**Authors:** Edis Hacılar, Pınar Yalınay Dikmen, Esme Ekizoğlu, Elif Ilgaz Aydınlar, Elif Kocasoy Orhan, Ferda Selçuk, Aynur Özge, Ayşenur Şahin, Tuba Erdogan Soyukibar, Bahar Taşdelen, Mustafa Ertaş, Betül Baykan

**Affiliations:** 1 Department of Neurology, Istanbul Faculty of Medicine, Istanbul University, Istanbul, Türkiye; 2 Department of Neurology, Acibadem University School of Medicine, Istanbul, Türkiye; 3 Department of Neurology, Burhan Nalbantoğlu State Hospital, Nicosia, Cyprus; 4 NOROM Neuroscience and Excellence Center, Ankara, Türkiye; 5 Department of Biostatistics and Medical Informatics, Mersin University Faculty of Medicine, Mersin, Türkiye

**Keywords:** agitation, cluster headache, cranial autonomic symptoms, photophobia, restlessness

## Abstract

**Background:**

ICHD-3 recognizes restlessness/agitation as an accompanying feature of cluster headache (CH). However, its phenotypic significance within CH remains uncertain. We compared patients with CH with (CHwR) and without restlessness (CHwoR) to characterize the clinical features and symptom burden associated with restlessness.

**Methods:**

We conducted a cross-sectional cohort study of Turkish patients with CH (*n* = 318) across tertiary neurology clinics, using a standardized clinician-administered questionnaire. We compared patients with CHwR versus CHwoR across clinical domains, applied multivariable logistic regression to identify independent correlates, and used Partitioning Around Medoids clustering (six pre-specified variables) to explore data-driven profiles.

**Results:**

There were no patients diagnosed with restlessness alone, without any autonomic symptoms. Restlessness co-occurred with cranial autonomic symptoms—particularly eyelid ptosis and sweating—and also with photophobia, nausea and vomiting. Independent correlates were photophobia (OR 2.91; 95% CI 1.65–5.14), sweating (1.96; 1.04–3.71), and eyelid ptosis (1.75, 1.01–3.03). Workplace burden tended to be greater in CHwR (work-day loss median 10 vs. 5 days; work-life impact 84.1% vs. 73.4%), with higher work-life impact and a borderline difference in work-day loss. Exploratory clustering showed a graded pattern of restlessness, highest in the earlier-onset/high-burden profile.

**Conclusion:**

In this tertiary-care cohort, restlessness was associated with a broader, symptom-rich CH phenotype characterized by both cranial autonomic and migrainous features and greater clinical burden. As no migraine comparison group was included, its diagnostic discriminatory value cannot be directly assessed. These findings suggest that restlessness may be better interpreted in the context of the overall CH phenotype rather than as an isolated diagnostic feature.

## Introduction

Cluster headache (CH) is a trigeminal autonomic cephalalgia characterized by strictly unilateral orbital/supraorbital/temporal pain lasting 15–180 min, occurring from once every other day up to eight times per day, and accompanied by ipsilateral cranial autonomic symptoms (CAS) (e.g., lacrimation, conjunctival injection, nasal congestion/rhinorrhea, eyelid edema, miosis/ptosis) or a marked sense of restlessness/agitation during attacks according to the International Classification of Headache Disorders-3 (ICHD-3) criteria (1). CAS are widely regarded as the clinical hallmark of CH; nevertheless, in contemporary cohorts a CH diagnosis may be assigned even when CAS are totally absent if restlessness/agitation is present, a position traceable to ICHD-II and retained in ICHD-3 (with small but non-zero proportions of patients meeting criteria without CAS) (2, 3). In CH series, the proportion of patients without CAS has been reported between 2.8 and 8.8%, with some diagnoses supported by the presence of restlessness during attacks (3, 4), leaving open how strongly restlessness should be weighted relative to CAS in nosology.

Restlessness is a behavioral phenomenon during CH attacks—patients often pace, rock, clutch the painful eye/temple, or even engage in self-injurious behaviors—and is consistently described as characteristic in Western cohorts (5, 6). The prevalence of restlessness in CH appears to vary across populations. Around 68–99% of patients in Western cohorts are reported as being restless (7, 8). In contrast, studies from East Asia describe lower rates and an uncoupling between the feeling of restlessness and visible restless behaviour: in Taiwan only 25.3% of patients reported restlessness, while in Japan about 69% acknowledged the sensation but only 43% showed overt behaviour, suggesting that both cultural factors and the mode of ascertainment influence reported prevalence (7, 9).

On the other hand, CH and migraine may show clinically meaningful overlap. Although distinguishable, they share unilateral pain, several pharmacologic triggers (e.g., glyceryl trinitrate, histamine, calcitonin gene-related peptide (CGRP)), and responses to triptans and anti-CGRP monoclonal antibodies, pointing to a shared trigeminovascular pathway (10). In migraine, the reported prevalence of ≥1 CAS ranges from about 3.1 to 82%, reflecting methodological and population heterogeneity (11). Conversely, in CH, migrainous features are common across cohorts; roughly photophobia (~34–78%), nausea (~27–53%), phonophobia (~15–49%), vomiting (~12–32%) (7, 12). Within this overlap, many reviews and cohort syntheses present “restlessness” as a practical differentiator: patients with CH commonly pace or cannot keep still, whereas patients with migraine often avoid movement (2, 5, 13–15). Nevertheless, the literature has not delineated phenotype by stratifying CH cohorts according to the presence of restlessness and comparing clinical features; consequently, it remains uncertain whether restlessness identifies a distinct clinical phenotype within CH. Accordingly, the aim of this study was to investigate whether restlessness defines a distinct CH phenotype by examining its associations with other CH symptoms, demographic characteristics, and indices of disease severity.

## Methods

### Participants and data collection

Patients were recruited from the tertiary neurology and headache outpatient clinics of the participating referral centres between January and June 2024, after providing written informed consent. All participants had a confirmed definite diagnosis of either episodic or chronic CH, established by a headache specialist according to the ICHD-3 criteria. Exclusion criteria were secondary CH, refusal to participate, illiteracy, and unstable medical or psychiatric conditions. This study represents a secondary analysis of data from the previously published work of the CLUES study group (16). As this was a secondary analysis, the sample size was fixed by data availability and no *a priori* power calculation was performed. A sensitivity analysis indicated a minimum detectable effect of approximately OR 2.1 (*α* = 0.05, 80% power) for a binary characteristic of intermediate prevalence; therefore, non-significant comparisons should be interpreted as inconclusive rather than as evidence of no difference. Data were complete for all reported variables, and no imputation was required.

To ensure diagnostic accuracy, all enrolled patients underwent re-evaluation by an experienced headache specialist, and their diagnoses were reconfirmed. Participants subsequently completed a semi-structured questionnaire, administered either through face-to-face interviews or telephone interviews conducted by the researchers. The questionnaire covered the following domains: (a) demographics, (b) family history and lifestyle habits, (c) comorbidities, (d) characteristics of CH attacks, (e) associated symptoms, (f) treatments received, and (g) patient-reported impact of CH on personal, social, and professional life. Even if nausea and other associated symptoms were present in only a subset of the attacks, they were still considered to be present. Restlessness was ascertained using a single binary, patient-reported item and was not severity-graded. Participants were asked: “Did you feel restless or have an urge to move during your attacks?”

### Statistical analysis

#### Descriptive statistics and between-group comparisons

Continuous variables were summarized as mean ± SD; categorical variables as counts (percentages). Between-group comparisons for continuous variables used Student’s *t* test when homoscedasticity was met (Levene’s test) and Welch’s *t* test otherwise; categorical variables were compared with Pearson’s χ^2^ test (or Fisher’s exact test when expected counts were <5). Analyses were performed in IBM SPSS Statistics (v22). Skewed continuous variables are additionally summarised as median (IQR) and compared with the Mann–Whitney *U* test. Univariable comparisons were not adjusted for multiple testing, as Table 1 was descriptive and hypothesis-generating, whereas the multivariable model constituted the primary inferential analysis. Statistical significance was assessed using two-sided tests with *p* < 0.05.

**Table 1:** Comparison of semographics, habits, and clinical characteristics between the groups.

Variables	CH without restlessness (*n* = 79)	CH with restlessness (*n* = 239)	Test statistic	*p*-value
Demographics				
Current age, years (mean ± SD)	50.3 ± 10.9	43.2 ± 10.3	t = 5.35	**<0.001**
Age at CH onset, years (mean ± SD)	32.4 ± 13.6	28.0 ± 11.2	t = 2.58	**0.011**
Age at CH diagnosis, years (mean ± SD)	38.4 ± 12.6	33.5 ± 10.3	t = 3.12	**0.002**
Male gender, *n* (%)	59 (74.7)	185 (77.4)	χ^2^ = 0.246	0.620
Habits				
Active or ex-Smoker, *n* (%)	58 (73.4)	192 (80.3)	χ^2^ = 1.690	0.194
Current passive smoking, *n* (%)	33 (41.8)	143 (59.8)	χ^2^ = 7.836	**0.005**
Alcohol use, *n* (%)	53 (67.1)	144 (60.3)	χ^2^ = 1.453†	0.484
Headache Characteristics				
Attack duration ≥ 3 h, *n* (%)	24 (30.4)	87 (36.4)	χ^2^ = 0.70	0.402
Average attacks per day (mean ± SD)	2.0 ± 1.6	2.2 ± 1.5	t = 0.98	0.326
Pain severity (VAS, 0–10) (mean ± SD)	9.3 ± 1.1	9.6 ± 1.1	t = 1.65	0.102
Chronic CH subtype, *n* (%)	28 (35.4)	85 (35.6)	χ^2^ = 0.00	1.000
Aura, *n* (%)	24 (30.4)	93 (38.9)	χ^2^ = 1.859	0.173
Side shift during cluster period, *n* (%)	9 (11.4)	12 (5.0)	χ^2^ = 3.91	**0.048**
Seasonal periodicity present, *n* (%)	51 (64.6)	183 (76.6)	χ^2^ = 4.42	**0.035**
Family history of CH, *n* (%)	11 (13.9)	22 (9.2)	χ^2^ = 1.422	0.233
Hospitalizations during CH period (mean ± SD)	1.2 ± 4.7	2.5 ± 6.8	U	0.037
Prior migraine diagnosis, *n* (%)*	33 (41.8)	91 (38.1)	χ^2^ = 0.280	0.596
Comorbid migraine diagnosis, *n* (%)	9 (11.4)	32 (13.4)	χ^2^ = 0.218	0.640
Autonomic Features				
Eyelid ptosis, *n* (%)	36 (45.6)	145 (60.7)	χ^2^ = 5.52	**0.019**
Rhinorrhea, *n* (%)	26 (32.9)	112 (46.9)	χ^2^ = 4.70	**0.030**
Sweating, *n* (%)	16 (20.3)	88 (36.8)	χ^2^ = 7.40	**0.007**
Conjunctival injection, *n* (%)	35 (44.3)	119 (49.8)	χ^2^ = 0.716	0.398
Lacrimation, *n* (%)	58 (73.4)	193 (80.8)	χ^2^ = 1.921	0.166
Nasal congestion, *n* (%)	50 (63.3)	174 (72.8)	χ^2^ = 2.580	0.108
Facial flushing, *n* (%)	13 (16.5)	63 (26.4)	χ^2^ = 3.202	0.074
Miosis, *n* (%)	11 (13.9)	55 (23.0)	χ^2^ = 2.982	0.084
Migrainous-Associated Symptoms				
Nausea, *n* (%)	17 (21.5)	83 (34.7)	χ^2^ = 4.81	**0.028**
Vomiting, *n* (%)	5 (6.3)	38 (15.9)	χ^2^ = 4.65	**0.031**
Photophobia, *n* (%)	25 (31.6)	143 (59.8)	χ^2^ = 18.93	**<0.001**

CH, cluster headache; CHwR, Cluster headache with restlessness; CHwoR, Cluster headache without restlessness; SD, standard deviation; VAS, visual analog scale, including misdiagnoses of CH besides comorbid migraine; ^†^compared across the three original response categories. Bold values show statistical significance.

#### Multivariable modeling

To identify factors independently associated with restlessness, we fitted a multivariable logistic regression using R (v4.2.1). Because the question concerned the attack phenotype, predictors were restricted to attack-level features: photophobia, nausea, vomiting, eyelid ptosis, sweating, rhinorrhea, pain severity using a visual analog scale (VAS), attack duration (<3 h vs. ≥ 3 h), and age at onset. Patient-level characteristics (sex, current age, smoking) lie outside this question and were not included; age at onset was entered as the sole age variable, as current age and age at diagnosis are downstream of it and strongly correlated (*r* = 0.57–0.77). Multicollinearity was assessed using variance inflation factors. Results are reported as odds ratios (ORs) with 95% confidence intervals (CIs) and Wald *p*-values. Model performance was summarized by the area under the receiver operating characteristic (ROC) curve (AUC) and McFadden’s pseudo-*R*^2^.

#### Unsupervised clustering

To identify data-driven patient subgroups, we performed a Partitioning Around Medoids (PAM) clustering analysis in R (v4.2.1) using a Gower’s distance matrix, which is optimal for mixed data types. The analysis was based on six clinical features chosen to represent the autonomic (eyelid ptosis, sweating, rhinorrhea) and migrainous (nausea, photophobia) domains together with age at CH onset; a deliberately small feature set was used because Gower–PAM solutions become unstable and dominated by sparse binary variables as dimensionality increases. The optimal number of clusters (*k* = 3) was determined by the average silhouette method. The quality of the final cluster solution was also evaluated by its average silhouette width. Because these features also entered the regression model, the clustering was regarded as exploratory and descriptive rather than as independent validation. Differences in the proportion of patients reporting restlessness across the resulting clusters were tested with a Pearson’s χ^2^ test.

## Results

We analyzed 318 patients diagnosed with CH: CHwR (*n* = 239) and CHwoR (*n* = 79) (Table 1). None of the patients in our study cohort were diagnosed solely on the basis of restlessness and agitation criteria without autonomic symptoms.

Patients with restlessness (CHwR) were younger (*p* < 0.001), had earlier CH onset (*p* = 0.011), and earlier diagnosis (*p* = 0.002), when compared with those without restlessness (CHwoR). Passive smoking was more frequent in the CHwR group (*p* = 0.005). Side shift during the cluster period was less common (*p* = 0.048), seasonal periodicity more frequent (*p* = 0.035), and hospitalizations were higher (*p* = 0.037) in the restlessness group. Other investigated variables did not show significant differences (Table 1).

Regarding symptoms, restlessness co-occurred with higher rates of eyelid ptosis (*p* = 0.019), rhinorrhea (*p* = 0.030), and sweating (*p* = 0.007); but other CAS did not differ significantly (Table 1). The “migrainous” features, assessed in this study, were also more frequent in the CHwR group: nausea (*p* = 0.028), vomiting (*p* = 0.031), and photophobia (*p* < 0.001).

Patients with CHwR showed greater work-life impact (84.1% vs. 73.4%; *p* = 0.034). Work-day loss was numerically higher in CHwR (median 10 vs. 5 days), although this difference was borderline using the Mann–Whitney *U* test (*p* = 0.050) (Table 2).

**Table 2:** Comparison of behavioral/psychosocial impact between the study groups.

Variables	CH without restlessness (*n* = 79)	CH with restlessness (*n* = 239)	Test statistic	*p*-value
Behavioral/psychosocial impact				
Suicidal Ideation, *n* (%)	19 (24.1)	69 (28.9)	χ^2^ = 0.689	0.406
Suicide Attempt, *n* (%)	1 (1.3)	5 (2.1)	χ^2^ = 0.219	0.640
Workday loss in last year (days; mean ± SD, median [IQR])	12.9 ± 20.2; 5 [0–15]	21.5 ± 44.9; 10 [1–25]	t = 2.34; U	0.020*/0.050**
CH period affected work life, *n* (%)	58 (73.4)	201 (84.1)	χ^2^ = 4.48	**0.034**
CH period affected family life, *n* (%)	55 (69.6)	184 (77.0)	χ^2^ = 1.73	0.189
CH period affected social life, *n* (%)	66 (83.5)	212 (88.7)	χ^2^ = 1.44	0.231

CH, cluster headache; SD, standard deviation; IQR, interquartile range; VAS, visual analog scale. Bold values show the results of *Independent Samples T-test and **Mann-Whitney U test.

Triptan use for acute treatment and valproate for prevention were more common in the CHwR group (*p* = 0.015 and *p* = 0.013, respectively), while there was no difference between these groups in their use of other acute and preventive therapies.

Variables were selected *a priori* based on clinical relevance and the study hypothesis. In multivariable logistic regression, photophobia (OR 2.91, 95% CI 1.65–5.14; *p* < 0.001), sweating (OR 1.96, 1.04–3.71; *p* = 0.039), and eyelid ptosis (OR 1.75, 1.01–3.03; *p* = 0.046) showed independent associations with restlessness, strongest for photophobia and modest for sweating and eyelid ptosis, whose confidence intervals approached unity; nausea, vomiting, rhinorrhea, pain severity, attack duration, and age at onset showed no significant association (Table 3). All variance inflation factors were ≤1.41, indicating no meaningful collinearity. Overall discrimination was modest (AUC 0.721; McFadden’s pseudo-*R*^2^ 0.111), indicating that the measured features identify associations rather than predict restlessness at the individual level. The adjusted effects and the ROC curve are shown in Figures 1a,b, respectively.

**Table 3:** Logistic regression analysis of predictors for restlessness.

Predictor variable	Coefficient (*β*)	Std. error (SE)	Odds ratio (OR)	95% CI for OR	*p*-value
Migrainous features					
Photophobia	1.068	0.291	2.91	1.65–5.14	<0.001*
Nausea	0.145	0.370	1.16	0.56–2.39	0.696
Vomiting	0.354	0.580	1.42	0.46–4.40	0.542
Autonomic features					
Sweating	0.674	0.326	1.96	1.04–3.71	0.039**
Eyelid ptosis	0.560	0.281	1.75	1.01–3.03	0.046**
Rhinorrhea	0.384	0.293	1.47	0.83–2.61	0.190
Other variables					
Pain severity (VAS)	0.087	0.117	1.09	0.87–1.37	0.457
Duration <3 h (vs. ≥3 h)	−0.188	0.300	0.83	0.46–1.50	0.531
Age at Onset	−0.016	0.012	0.98	0.96–1.01	0.160
*(Intercept)*	−0.306	1.126	0.74	0.08–6.70	0.786

Significance codes: *p < 0.001, **p < 0.05. Bold values show statistical significance.

**Figure 1:**
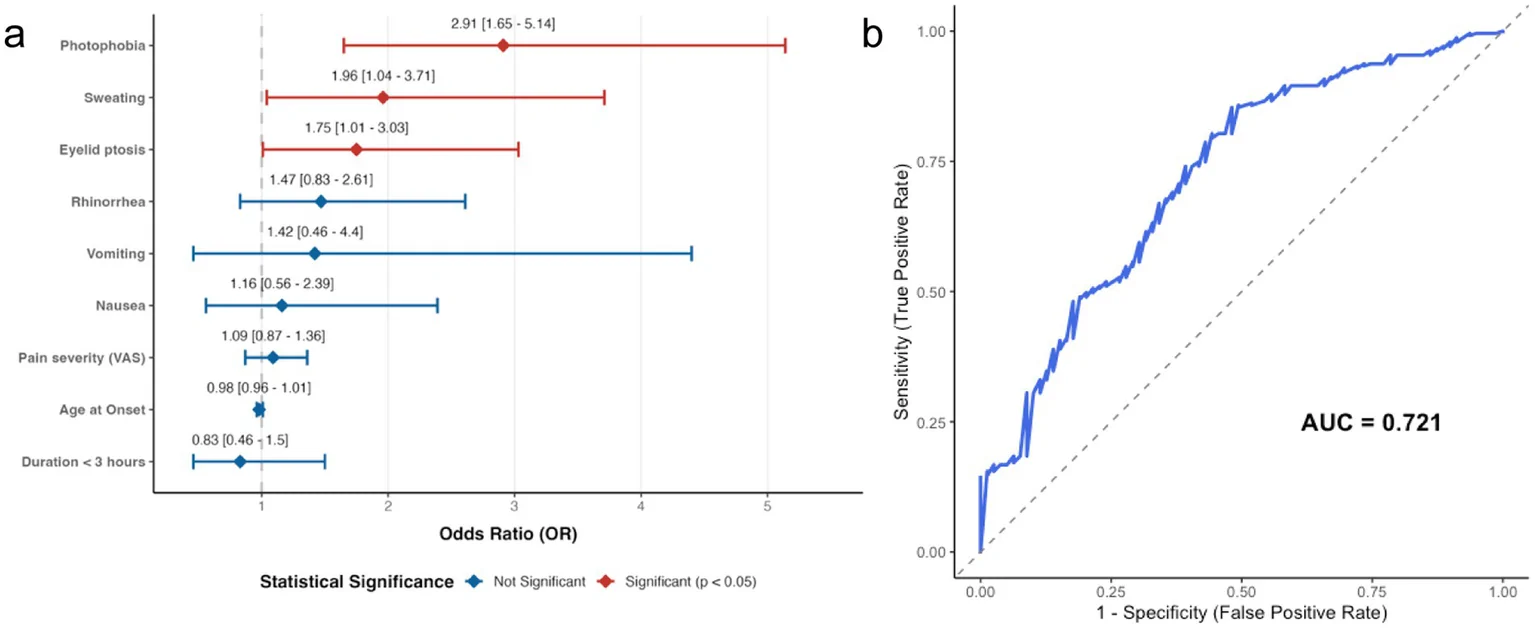
**(a)** Forest plot of adjusted odds ratios (OR) and 95% confidence intervals (CI) for predictors of restlessness from the multivariable logistic regression model. **(b)** The corresponding Receiver Operating Characteristic (ROC) curve, (AUC = 0.721).

Unsupervised PAM clustering with Gower’s distance on six selected features (nausea, photophobia, eyelid ptosis, sweating, rhinorrhea, and age at CH onset) yielded three exploratory profiles with differing burden and restlessness rates: (1) “Higher-Burden/Earlier-Onset” profile (*n* = 83; restlessness 95.2%), where clinical burden reflects the highest cumulative count of concurrent autonomic and migrainous symptoms, (2) “Moderate-Burden/Intermediate-Onset” profile (*n* = 143; 92.3%), and (3) “Lower-Burden/Later-Onset” profile (*n* = 92; 30.4%). The differences in restlessness across clusters were highly significant (χ^2^(2) = 138.9, *p* < 0.001). Cluster quality was weak (average silhouette width = 0.26), indicating a graded spectrum rather than discrete subgroups. Detailed characteristics are presented in Figure 2 and Supplementary Table 1.

**Figure 2:**
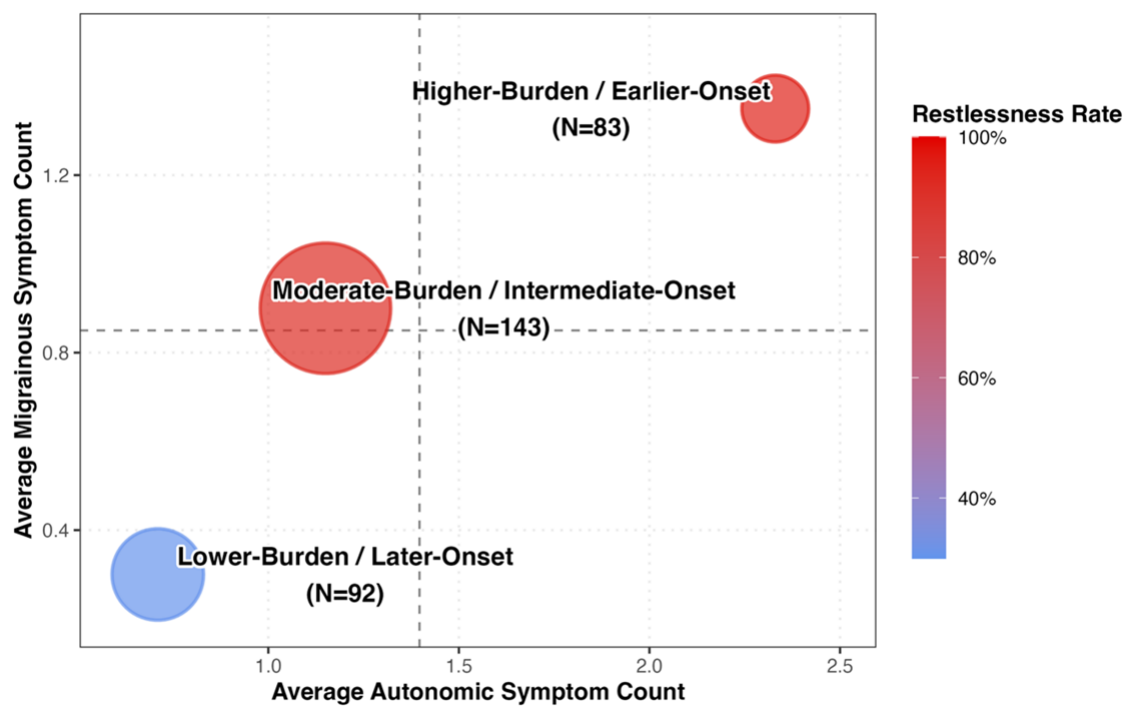
Clinical Profiles of Cluster Headache Subgroups identified in Cluster Analysis. Patient clusters were identified through a PAM analysis of 6 key clinical features. The position of each cluster is determined by its average autonomic symptom count (x-axis) and average migrainous symptom count (y-axis). The size of each bubble is proportional to the number of patients (*N*) in the cluster, and the color corresponds to the rate of restlessness, with darker red indicating a higher prevalence. The dashed horizontal and vertical lines indicate the mean values for the migrainous and autonomic symptom counts across all three clusters.

## Discussion

In this cohort of 318 patients with CH, restlessness during attacks clustered with a higher burden of both CAS—especially eyelid ptosis and sweating—and migrainous features (nausea, vomiting, photophobia). Photophobia, and more modestly ptosis and sweating, remained independent associates of restlessness in multivariable analysis, and patients with restlessness showed greater work-life impact and higher triptan use. An exploratory clustering analysis further showed graded restlessness rates across three clinical profiles, highest in an earlier-onset, higher-burden subgroup. Overall, restlessness was associated with a broader symptom burden and greater clinical burden, although the cross-sectional design does not permit causal or temporal inference.

Although CAS define CH, non-CAS CH is reported in 2.8–8.8% when ICHD-3 criteria permit diagnosis with restlessness/agitation (3, 4). In the largest prospective series, 47.9% of non-CAS patients reported restlessness yet diagnosis rarely depended on it and the non-CAS phenotype was reported to be milder (3). That none of the patients in our cohort received a diagnosis of CH on the basis of restlessness/agitation alone, together with the small proportions reported in previous series (3, 4), suggests that this subcriterion—although biologically plausible and weighted in ICHD-3 as equivalent to cranial autonomic symptoms—may be less frequently decisive in clinical practice than its current weighting might imply. Notably, tertiary clinics are enriched for atypical and diagnostically challenging presentations, where such patients might be expected to be encountered. The absence of such cases in our cohort suggests that this subcriterion may be infrequently decisive in tertiary clinical practice, although referral and regional effects cannot be excluded. These observations warrant further evaluation in population-based and multicentre studies.

In migraine, CAS are typically bilateral, milder and less tightly time-locked than in CH, and CAS-positive migraine cohorts tend to have more severe and more frequent attacks—a pattern consistent with our observation that the “migrainous” profile in CH carried greater disease burden (11, 17). Conversely, “migrainous” features are common in CH across cohorts (photophobia, nausea, vomiting), and our findings align with previously published ranges (7, 12). Expert opinion, reviews and clinical texts have treated restlessness as a practical discriminator (2, 5, 13–15), yet this rule may not be absolute in heterogeneous clinical settings. In our cohort, restlessness co-occurred with higher rates of migrainous features. Because no migraine comparison group was included, our data cannot quantify how well restlessness distinguishes CH from migraine. Rather, its co-occurrence with migrainous features highlights the importance of interpreting restlessness within the broader clinical phenotype.

Mechanistic data regarding restlessness in CH converge on a network model anchored by the hypothalamus and linked brainstem and limbic circuits. Shared trigeminovascular activation projects to the trigeminal cervical complex with downstream connections to thalamus, hypothalamus, basal ganglia, periaqueductal gray, and locus coeruleus, providing routes for coordinated nociceptive, autonomic, and arousal responses (10, 18–20). Neuroimaging studies support cortical–hypothalamic–brainstem interconnections that may switch between out-of-bout and in-bout states and ignite both the trigeminovascular system and the trigeminal autonomic reflex—consistent with concurrent CAS and the urge to move (restlessness) during attacks of CH (18). This central urge has recently been mapped to a specific brainstem-cortical loop; ictal fMRI data demonstrate that restlessness in CH is underpinned by enhanced locus coeruleus–substantia nigra functional coupling paired with a failure of top-down prefrontal inhibition (19). No imaging or neurophysiological data were acquired in the present study; this network model derives from published work and is offered as an interpretative framework rather than a demonstrated mechanism (13, 20). Our data showed that the prevalence of restlessness was uniform across the episodic and chronic subtypes (*p* = 1.000), suggesting that the expression of restlessness is independent of the temporal pattern of the disease. Brainstem nuclei and their connections to the superior salivatory nucleus and hypothalamus help explain CAS in migraine as well as CH, reinforcing that autonomic phenomena are not diagnosis-specific when these networks are strongly engaged (17, 21). Limbic participation may also contribute to the behavioral phenotype. Increased amygdala metabolism has been linked to anxiety and greater disability in CH (22). Psychiatric comorbidities, including anxiety and depression, are frequently observed in CH and independently contribute to functional impairment and disease severity (23).

In our cohort, restlessness was more common among younger patients and was associated with greater symptom and work-life burden, although the cross-sectional design precludes conclusions regarding directionality. In our previous study distinct clusters were also identified (24), but family history did not differentiate the groups here. Passive smoking was also more frequent in the restlessness group; given the established link between cluster headache and tobacco exposure, this may reflect shared household environments rather than a specific association with restlessness and requires confirmation.

A role for inflammation in CH has also been suggested but has not yet been linked to distinct phenotypes (25). Furthermore, we hypothesized that restlessness might serve as a predictor for treatment response, either in the selection of acute or preventive options. However, our results could not support this hypothesis. Triptan and valproate use were more frequent in the restlessness group; because treatment utilisation reflects severity, care-seeking and prescriber preference rather than biology, we interpret this as a further marker of burden rather than an independent association.

In our data, restlessness was not associated with longer attack duration but co-occurred with greater migrainous and autonomic symptom burden. Reports on CH lacking CAS reveal lower anxiety/depression scores and attenuated pain severity, supporting the view that the non-CAS phenotype is milder and that restlessness alone is an incomplete marker of disease expression (3, 4). Nosologically, the growing recognition of migraine with CAS and of CH with migrainous symptoms argues for contextualising restlessness within the total clinical picture rather than privileging it as a substitute for CAS (3, 4, 11, 17, 26); “side-locked migraine” further illustrates this thin boundary (27).

### Strengths and limitations

Key strengths of this study include its large, multicenter cohort of patients with headache-specialist–confirmed CH diagnoses and the detailed assessment of clinical, cranial autonomic, migrainous, behavioural, and functional characteristics. To our knowledge, this is the first study to stratify a CH cohort according to the presence of restlessness and to examine its clinical associations in detail. The primary inferential analysis was based on multivariable logistic regression, while the clustering analysis was used in an exploratory and descriptive manner to further characterise symptom patterns rather than as an independent validation of the regression findings.

Several limitations should be acknowledged. First, the cohort included only patients with confirmed CH and did not include a migraine comparison group; therefore, the present findings describe associations within CH and cannot be used to estimate the sensitivity, specificity, or diagnostic discriminatory accuracy of restlessness for distinguishing CH from migraine. Second, the cross-sectional design precludes causal or temporal inference, and the data cannot determine whether restlessness contributes to greater clinical burden, results from it, or reflects a shared underlying process. Third, recruitment from tertiary referral headache clinics may over-represent patients with more severe, refractory, or diagnostically complex disease, limiting generalisability to community-based populations. Differences in admission thresholds between centers may also have contributed to the observed difference in hospitalisation rates independently of disease severity.

Fourth, retrospective ascertainment introduces the possibility of recall bias, particularly for quantitative variables such as work-day loss. Restlessness was assessed using a single, unvalidated, patient-reported binary item and was not severity-graded; therefore, some degree of misclassification cannot be excluded. In addition, interview mode was not recorded at the individual level, so any potential effect of telephone versus face-to-face assessment on symptom reporting could not be examined.

Fifth, because the sample size was fixed by the available cohort, the study had limited power to detect small associations, particularly in the smaller CHwoR group. Non-significant comparisons should therefore be interpreted as inconclusive rather than as evidence of no difference, and borderline univariable findings should be regarded as exploratory. Finally, the average silhouette width of the clustering solution was weak, indicating that the identified profiles are better interpreted as a graded clinical spectrum rather than as discrete subgroups. Because the variables used for clustering also entered the regression model, the clustering analysis should be regarded as exploratory and descriptive rather than as independent validation.

Prospective, population-based, and multicenter studies are needed to confirm these findings, assess the reproducibility of the identified symptom patterns, and further clarify the clinical significance of restlessness across different cluster headache populations.

## Conclusion

Within CH, restlessness was associated with a broader symptom-rich phenotype characterized by greater migrainous and cranial autonomic symptom burden, with photophobia showing the strongest independent association. As no migraine comparison group was included, the diagnostic discriminatory value of restlessness cannot be directly assessed. These findings suggest that restlessness may be better interpreted in the context of the overall CH phenotype rather than as an isolated diagnostic feature.

## Data Availability

The raw data supporting the conclusions of this article will be made available by the authors, without undue reservation.
