# Supplementary material for: Restlessness in cluster headache: unveiling the role of its key associations and clinical implications from a cohort study
Source: Front Neurol. 2026 Oct 5;17:1946721. doi: 10.3389/fneur.2026.1946721 (PMC13677482; doi:10.3389/fneur.2026.1946721)
Supplement: Supplementary file 1 [file Table_1.docx]

**Supplementary Table 1:** Characteristics of Patient Clusters from Gower-PAM Analysis (k=3)

| **Clinical Profile** | **N** | **Avg. Migrainous Symptoms** | **Avg. Autonomic Symptoms** | **Avg. Age at Onset** | **Restlessness Rate** |
| --- | --- | --- | --- | --- | --- |
| **Higher-Burden/Earlier-Onset** | 83 | 1.35 | 2.33 | 24.3 | 95.2% |
| **Moderate-Burden/Intermediate-Onset** | 143 | 0.90 | 1.15 | 29.4 | 92.3% |
| **Lower-Burden/Later-Onset** | 92 | 0.30 | 0.71 | 32.9 | 30.4% |
